# The Normal and Pathological Histology of the Human Eye and Eyelids

**Published:** 1887-03

**Authors:** 


*The Normal and Pathological Histology of the Human Eye and Eyelids.* By C. FRED. POLLOCK, M.D., F.R.S. Eng. With 230 original Drawings. London: J. & A. CHURCHILL.

This book mainly consists of excellent lithographic plates very carefully copied by the author from microscopic specimens prepared by him during several years of continuous research. A short description accompanies each plate, so that no one can fail to understand its meaning; and 160 pages of well-written descriptions of the various structures composing the eye, under conditions of health and of disease, assist in forming a volume which will be found of practical use in the eye-work of the practitioner as well as in the study of the ophthalmic surgeon.

The rational treatment of diseases of the eye can only be based upon a competent knowledge of the minute structure of its constituent parts, as well as of their anatomical arrangement. A good clinician is likely also to be a competent microscopist; but it is a great gain to possess accurate representations of so complete a set of specimens as this volume includes.

The first part of the book describes, concisely but thoroughly, the various structures composing the eye, arranged in anatomical sequence: each one is considered—1st, in its normal histology; 2nd, under its more common pathological alterations.

The order of the illustrations found in the latter part of the book agrees as closely as possible with that of the text, and the one part should be read in reference to the other.

One hundred plates contain 230 excellent lithographic copies of the tissues, in health and in disease, and scarcely any condition that could be expected is absent; amongst them are sclerotitis, episcleritis, Descemetitis, lymphatics of choroid, pars ciliaris retinæ, cystic degenerations of ora serrata, and a series illustrating the development of the eye.

The book is based upon a study of the most important monographs of each subject, and can be highly recommended as complete of its kind. It covers the ground occupied by Pagenstecher's and Genth's atlas, and will be found more generally useful than the larger work, though it does not fully replace it.

